# Prevalence and Predictors of Post-Acute COVID-19 Symptoms in Italian Primary Care Patients

**DOI:** 10.1177/21501319231222364

**Published:** 2024-01-03

**Authors:** Andreana Foresta, Luisa Ojeda-Fernández, Claudia Augurio, Cecilia Guanziroli, Mauro Tettamanti, Giulia Macaluso, Paolo Lauriola, Alessandro Nobili, Maria Carla Roncaglioni, Marta Baviera

**Affiliations:** 1 Istituto di Ricerche Farmacologiche Mario Negri IRCCS, Milan, Italy; 2 ATS Brianza, Monza, Italy; 3 ASST Lariana, Como, Italy; 4 International Society of Doctors for Environment (ISDE), Rete Italiana Medici Sentinella per l’Ambiente, Geneva, Switzerland

**Keywords:** COVID-19, SARS-CoV-2, post-acute COVID-19, general practice, survey

## Abstract

**Background:**

Despite all the progress in the management of acute COVID-19, it is still not clear why some people continue to experience symptoms after recovery. Using data from a self-administered online survey, we assessed the prevalence and predictors of post-acute COVID-19 in an unselected population followed by GPs.

**Methods:**

Patients ≥18 years with a confirmed COVID-19 diagnosis were included. The survey collected information on demographics, risk factors, COVID-19 course and symptomatology. Fatigue and Quality of Life questionnaires were also administered. Descriptive statistics were used to describe patients’ characteristics, stratified as acute and post-acute COVID-19. Logistic regression models were used to assess the association between clinical characteristics and post-acute COVID-19.

**Results:**

A total of 1108 surveys were analyzed. Nearly 29% of patients reported post-acute COVID-19. The more persistent symptoms were fatigue, memory and concentration impairment. Adjusted Odds Ratio (OR) showed a significantly higher probability of post-acute COVID-19 for women compared to men (OR 1.9, 95% CI 1.4-2.5), for age >50 years than ≤50 years (OR 1.6, 95% CI 1.2-2.2), for BMI > 25 compared to BMI ≤ 25 (OR 1.6, 95% CI 1.1-2.1) and those with autoimmune diseases, compared to those without (OR 1.8 95% CI 1.1-2.9). In addition, a significant association was found with COVID-19 hospitalization, anxiety and allergies. We found that post-acute COVID-19 patients showed a higher fatigue and a worst quality of life.

**Conclusions:**

These findings suggest the need for tailored personalized strategies to improve the management of patients with post-acute COVID-19.

## Introduction

Since the spread of Coronavirus disease 2019 (COVID-19), a lot of progress have been made in the characterization and management of the acute phase of the disease.^1^ However, recovery from acute COVID-19 infection is not yet fully understood. After recovery from their acute illness, some people continue to experience the symptoms of the disease, affecting many systems including the respiratory, cardiovascular, neurological, gastrointestinal, and musculoskeletal systems.^2^

Moreover, the array, the manifestation and the duration of these symptoms vary, regardless of the initial severity of the disease.^3^

Post-Acute COVID-19, also known as Long COVID-19,^4^ is used to describe persistent symptoms and/or delayed or long-term complications more than four weeks from the onset of symptoms.^5^ Accumulated data is showing that the most prevalent post-acute COVID-19 symptoms are weakness, general malaise, fatigue and concentration impairment.^2,6^ The main risk factors for these sequelae seem to be female sex, age, obesity, smoking, and preexisting clinical conditions such as chronic obstructive pulmonary disease, fibromyalgia, and anxiety.^2,7^ Several studies have been conducted to understand this phenomenon better, but they differ widely in terms of study populations and follow-up periods.^2,6^ Most of studies on COVID-19 symptoms focused on hospitalized patients or those attending outpatient clinics^8-12^ so the selection of these specific settings implies non-generalizability of the results to the population with COVID-19.

Primary care physician is the patient’s first point of contact with the healthcare system and provides curative, rehabilitative, and palliative services throughout the patient’s life, thus general practitioners (GPs) can provide personalized, focused interventions for people recovering from COVID-19.^13-16^ However, data from general practice are scanty and only few studies have been conducted on self-reported data from unselected cohorts, estimating the prevalence and the predisposition for post-acute COVID-19 in real-world settings.^17-22^

Using data from a self-administered online survey, we assessed the prevalence and predictors of post-acute COVID-19 in an unselected Italian population, one of the most affected in Europe, followed by GPs since the beginning of pandemic. Additionally we investigated the impact of post-acute COVID-19 on health-related quality of life.

## Methods

### Study Design and Recruitment

This was a national, cross-sectional, observational cohort study, promoted by the Laboratory of Cardiovascular Prevention at the Istituto di Ricerche Farmacologiche Mario Negri IRCCS, and conducted in collaboration with an extensive network of GPs. From October 2021 to December 2022, the GPs were asked to invite all their patients with a confirmed COVID-19 diagnosis, from the beginning of the pandemics, to participate in an online survey on the persistence of COVID-19 symptoms. The last survey was received by 17/12/2022.

A total of 333 GPs were involved (The complete list of participating GPs is available in Supplemental Annex 1).

Participants were invited by e-mail or a WhatsApp message to take part voluntarily in this online survey. The invitation gave information on the purpose of the survey, data handling and a link to the online survey.

All individuals ≥18 years, on the lists of the GPs, with a confirmed COVID-19 diagnosis since the beginning of the pandemic were eligible. Diagnosis could be performed either by the GPs, accredited laboratories or at the pharmacies. Patients could participate the study whether they were hospitalized or not during COVID-19.

### Survey

The survey was developed by an interdisciplinary research team (researchers, statisticians and GPs). Before completing the survey, participants were asked to click the “I agree” button on the online informed consent form. The survey comprised the following topics: (1) demographics and risk factors, (2) self-reported comorbidities, (3) acute phase of the disease (eg, date of diagnosis, hospitalization status, oxygen needs in hospital), (4) symptomatology related to COVID-19, its duration and treatment. To assess the impact of COVID-19 symptoms on lifestyles, 2 validated questionnaires on Fatigue and Quality of Life were included in the online survey. The Fatigue Impact Scale (FIS) comprises dimensions related to physical, cognitive, psychosocial functioning, occupational, and social functioning. The 5Q-5D-3L investigates the following 5 dimensions, each describing a different aspect of health: mobility, self-care, usual activities, pain/discomfort, and anxiety/depression.^23,24^ The response formats were multiple-choice answers, yes-or-no answers, and free text entries. The completion of the online survey took about 15 minutes. The final version of the survey can be found in Supplemental Material (Supplemental Annex 2).

### Ethics

The study protocol was approved by the lead Ethics Committee (Fondazione IRCCS Istituto Neurologico Carlo Besta of Milan). Online informed consent and Data Protection sheet were obtained from all the participants.

Personal data have been processed in compliance with the provisions set out in EU Regulation 2016/679 (the “GDPR”) and in Legislative Decree N. 196/2003 (Personal Data Protection Code, and then the Legislative Decree No. 101/2018).

### Statistical Analysis

Continuous variables are shown as mean ± standard deviation (SD), and categorical variables are reported as frequencies and percentages. Descriptive statistics were used to describe demographic and key clinical characteristics of the study population, also stratified by duration of symptoms (0-4 weeks vs >4 weeks).

Data were then aggregated for the following periods: within 4 weeks after the infection (“acute COVID-19”), >4 weeks after the infection (“post-acute COVID-19”). The distribution of the frequency of symptoms was also reported according to age (18-40; 41-60 and >61 years) and sex, in both groups.

Logistic regression models were used to assess the association between clinical characteristics and post-acute COVID-19, taking into account all the variables reported in Tables 1 and 2.

**Table 1.** Baseline Characteristic of the Study Population.

Variables no, (%)	Participants no. (1108)
Age (years), mean ± SD	48.00 ± 15.40
Sex (female)	718 (64.80)
BMI mean ± SD	23.85 ± 4.77
Region	
Lombardy	767 (69.2)
Apulia	75 (6.8)
Latium	35 (3.2)
Other regions	321 (20.8)
Smoking	
Current	167 (15.07)
Never	791 (71.39)
Previous	150 (13.54)
History of comorbidities	
Cardio-cerebrovascular diseases	51 (4.60)
Hypertension	177 (15.97)
Hypercholesterolemia	252 (22.74)
Diabetes mellitus	40 (3.61)
Anemia	86 (7.76)
Chronic respiratory diseases	67 (6.05)
Gastrointestinal diseases	96 (8.66)
Liver diseases	17 (1.53)
Kidney diseases	19 (1.71)
Autoimmune diseases	82 (7.40)
Allergies	311 (28.07)
Tumor	51 (4.60)
Anxiety	240 (21.66)
Depression	63 (5.69)
COVID-19 related information	
Hospitalization during acute COVID-19	42 (3.80)
Oxygen need in hospital	34 (80.95)
ICU admission	11 (26.19)
Intubation	8 (19.04)
Age of hospital patients (years), mean ± SD	61.76 ±14.82
Discharged to no (%)	
Home	32 (76.19)
Care facility	10 (23.81)
Symptoms ≤ 4 weeks, no (%)	
No symptoms	39 (3.5)
1 symptom	49 (4.4)
≥2 symptoms	1020 (92.06)
Symptoms > 4 weeks, no (%)	
No symptoms	788 (71.12)
At least 1 symptom	320 (28.9)
1 symptom	105 (32.8)
≥2 symptoms	215 (67.2)
Acute COVID-19 group, no (%)	749 (67.59)
Post-acute COVID-19 group, no (%)	320 (28.9)

**Table 2.** Baseline Characteristics of the Acute COVID-19 Group and Post-Acute COVID-19 Group.

Variables no, (%)	Acute COVID-19 *N* = 749	Post-acute COVID-19 *N* = 320	*P*-value
Age (years), mean ± SD	46.82 ± 15.43	50.23 ± 14.56	.0008
Sex (female)	455 (60.75)	240 (75.00)	<.0001
BMI mean ± SD	23.60 ± 4.56	24.46 ± 5.20	.0074
Smoking			
Current	112 (14.95)	47 (14.69)	.9936
Never	537 (71.70)	230 (71.88)	
Previous	100 (13.35)	43 (13.44)	
History of comorbidities			
Cardio-cerebrovascular diseases	34 (4.54)	15 (4.69)	.9155
Hypertension	115 (15.35)	55 (17.19)	.4528
Hypercholesterolemia	157 (20.96)	84 (26.25)	.0581
Diabetes mellitus	23 (3.07)	12 (3.75)	.5676
Anemia	55 (7.34)	27 (8.44)	.5381
Chronic respiratory diseases	37 (4.94)	27 (8.44)	.0273
Gastrointestinal diseases	55 (7.34)	41 (12.81)	.0042
Liver diseases	9 (1.20)	8 (2.50)	.1202
Kidney diseases	12 (1.60)	7 (2.19)	.5017
Autoimmune diseases	43 (5.74)	39 (12.19)	.0003
Allergies	191 (25.50)	112 (35.00)	.0016
Tumor	32 (4.27)	16 (5.00)	.5988
Anxiety	139 (18.56)	97 (30.31)	<.0001
Depression	34 (4.54)	28 (8.75)	.0070
COVID-19 related information			
Hospitalization during acute COVID-19	14 (1.87)	27(8.43)	<.0001
Oxygen need in hospital	13 (92.86)	21 (77.78)	.2237
ICU admission	7 (50.00)	4 (14.81)	.0159
Intubation	4 (28.57)	4 (14.81)	.2919
Age of hospitalized patients (years), mean ± SD	65.85 ± 16.30	60.70 ± 13.10	.2789
Discharged to no (%)			
Home	11 (78.57)	21 (77.78)	.9536
Care facility	3 (21.43)	6 (22.22)	

Fatigue Impact Scale and 5Q-5D-3L Questionnaires reporting the frequencies of each response for each group were analyzed.

All analyses were done using SAS version 9.4 (SAS Institute, Cary, NC, USA).

## Results

The flow-chart of the study is set out in Supplemental Figure 1S. In total, 1748 subjects responded to the survey. Of these, 27 (1.5%) were excluded because they did not meet the eligibility criteria. Another 613 (35.1%) individuals were removed because they did not complete the survey, leading to a data set of 1108 (63.4%) surveys analyzed. Symptomatic subjects were stratified in 2 subgroups according to the duration of symptoms: acute COVID-19 (at least 1 symptom within 4 weeks) and post-acute COVID-19 (at least 1 symptom after 4 weeks). We identified 749 acute COVID-19 patients and 320 post-acute COVID-19 patients (28.9% of the total population), 32.8% of whom reported the persistence of only 1 symptom, and 67.2% 2 or more (Table 1).

The baseline characteristics of the study cohort are shown in Table 1. The majority of participants were female (718; 64.8%); the mean age was 48 years, the mean Body Mass Index (BMI) 23.8 ± 4.8 and 791 (71.4%) participants were non-smokers. Participants came from almost all Italian Regions, with the highest numbers of participants being from Lombardy, Apulia and Latium. The most common pre-existing clinical conditions included allergies, hypercholesterolemia and anxiety. Only 42 (3.8%) of participants had been hospitalized during the acute phase of the disease, and 11 (26.2%) of them were admitted to intensity care unit (ICU), of which 8 patients (19.0%) were intubated.

A total of 39 (3.5%) subjects were asymptomatic during the acute phase of the disease, while 49 (4.4%) experienced only 1 symptom and 1020 (92.1%) 2 or more symptoms within 4 weeks. The COVID-19 symptoms most frequently experienced were fatigue (73.8%), fever (60.6%), and cough (60.9%), closely followed by arthralgia, myalgia, and headache.

The prevalence of each symptom and their mean and median duration, both during the whole study period and after 4 weeks is reported in Supplemental Table 1S.

The clinical characteristics of the acute-COVID-19 group and post-acute COVID-19 group are presented in Table 2. Post-acute COVID-19 subjects were older, more female, more overweight, and in general presented more preexisting comorbidities, than the acute COVID-19 subjects. The post-acute COVID-19 group also had a significantly higher proportion of subjects hospitalized during the acute phase, compared to the acute COVID-19 group (8.4 vs 1.9%, <0.0001).

**Figure 1.**
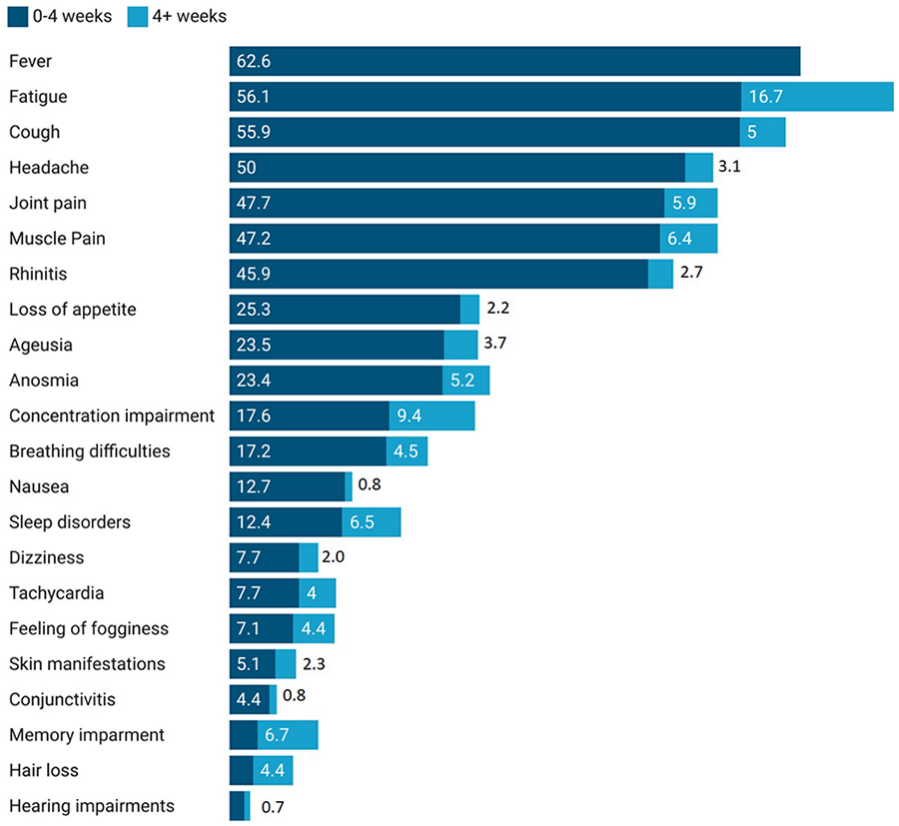
Prevalence of COVID-19 symptoms (percentages), in the period between 0 and 4 weeks (Blue bar) and after 4 weeks (Light blue bar).

The frequencies of each symptom in the acute COVID-19 group and post-acute COVID-19 group are reported in Figure 1. Fever occurred only in acute COVID-19, and the more persistent symptoms in post-acute COVID-19 were fatigue, memory impairment and concentration impairment.

**Figure 2.**
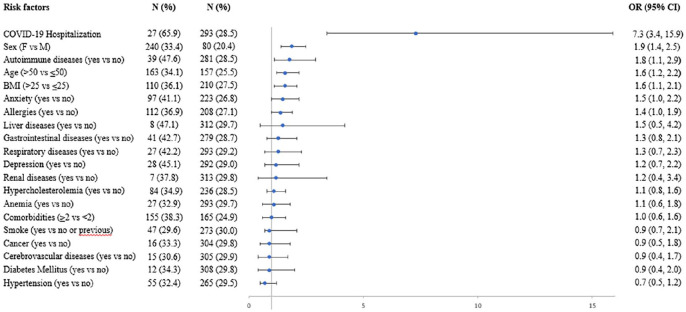
Multivariable logistic regression analysis of the association of post-acute COVID-19 with patients’ characteristics.

Age and sex analysis indicated that the acute COVID-19 group had a higher proportion of women aged 18 to 40 (26.2%) and 41 to 60 years (23.4%). The post-acute COVID-19 group, the proportions of women increased for all age classes considered, with the highest proportion in 41 to 60 age class (33.1%) (Figure 2S).

Adjusted OR for post-acute COVID-19 according to baseline characteristics are reported in Figure 2. Women had a significantly higher probability of manifesting COVID-19 related symptoms over 4 weeks (OR 1.9, 95% CI 1.4-2.5) than men. There was also a significantly higher odds of persistent symptoms with age >50 years, compared to age ≤50 years (OR 1.6, 95% CI 1.2-2.2), and BMI > 25 compared to BMI ≤ 25 (OR 1.6, 95% CI 1.1-2.1), COVID-19 hospitalization (OR 7.3, 95% CI 3.4-15.9), and autoimmune diseases (OR 1.8, 95% CI 1.1-2.9). Other pre-existing clinical conditions associated with a significantly higher probability of persistent symptoms were anxiety (OR 1.5, 95% CI 1.0-2.2) and allergies (OR 1.4, 95% CI 1.0-1.9).

### Fatigue Impact Scale Questionnaire

**Figure 3.**
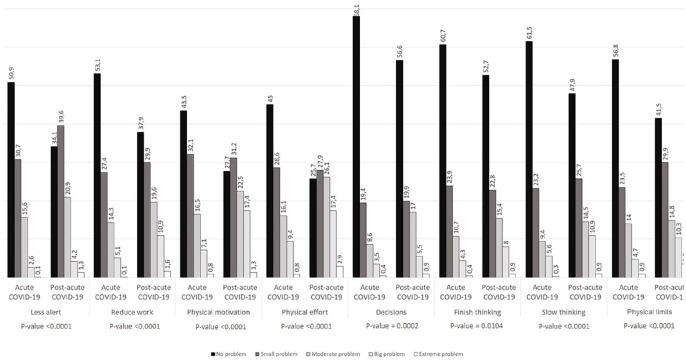
Percentages of acute COVID-19 and post-acute COVID-19 subjects for the different domains of the Fatigue Impact Scale questionnaire.

Answers to the FIS questionnaire were available for 1031 participants. A significantly higher proportion of patients in post-acute COVID-19 group, with respect to those in acute COVID-19 group, reported that fatigue caused them problems in being alert, finishing their workload, making hard and prolonged physical effort (all *P* < .0001) and making decisions (Figure 3). More than 17% of participants in the post-acute COVID-19 group reported greater problems in physical motivation (17.4 vs 7.1%, *P* < .0001) and in maintaining physical effort (17.4 vs 9.4%, *P* < .0001), than the acute COVID-19 group.

### 5Q-5D-3L Questionnaire

**Figure 4.**
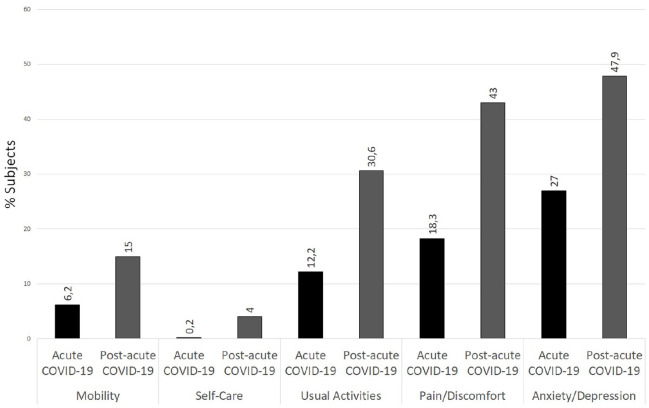
Percentages of acute COVID-19 and post-acute COVID-19 subjects reporting limitations on each domain of the EQ-5D-5L questionnaire.

Answers to the 5Q-5D-3L questionnaire are available for 1013 participants. A significantly higher proportion of participants in the post-acute COVID-19 group reported limitations in mobility and in their ability to carry out their usual activities (all *P* < .0001). A higher proportion of participants in the post-acute COVID-19 group reported moderate or high pain than those in the acute-COVID-19 group (43 vs 18.3%, *P* < .0001). In addition, almost 50% of participants in the post-acute COVID-19 group reported a moderate or higher level of anxiety or depression that those in the acute-COVID-19 group (47.9 vs 27%, *P* < .0001) (Figure 4).

## Discussion

In our cohort of more than 1000 primary care patients with a confirmed diagnosis of COVID-19, the symptoms most frequently reported by participants were fatigue, fever, and cough, consistent with the literature reporting similar symptoms in hospitalized and non-hospitalized patients.^8,9,25,26^

Of note, 96.4% (1069 out of 1108) of the participants experienced symptomatic COVID-19, of which 320 (29.9% of 1069) reported the persistence of at least 1 symptom for longer than 4 weeks. This is in line with a study by Bliddal et al^17^ in which 36% of patients reported the persistence of symptoms. However, in a longitudinal study on 180 non-hospitalized COVID-19 patients Petersen et al^21^ found that more than 50% experienced at least 1 symptom persisting after the acute phase of the disease. This proportion is definitely higher than that observed in our population and in the Danish study.^17^

In our population, the most persistent symptoms were fatigue, concentration impairment, and memory disorders. This is consistent with the results of other studies where participants directly reported symptoms, with fatigue being the most persistent symptom.^17,21,26-31^ In addition to fatigue, loss of smell and taste were the most persistent symptoms as reported in a Danish study,^21^ in 2 Norwegian studies,^20,25^ and in a recent population based study conducted in Michigan.^32^ These studies showed that the prevalence of loss of smell and taste ranged between 10 and 14%, while in our study, 4% of participants reported persisting taste and smell alterations beyond 4 weeks. In addition, the 2 Norwegian studies also reported arthralgia and dyspnea as 2 of the most persistent symptoms, with prevalence rates between 9 to 16%, while the prevalence in our population was lower at 5.9% for arthralgia and 4.5% for breathing difficulties.^20,25^ The higher prevalence of loss of smell, loss of taste, arthralgia, and dyspnea after the acute phase observed in previous publication might be influenced by the time of inclusion since participants were infected during the first pandemic wave.

As emerged also by ours and other results, post-acute COVID-19 conditions often involved a range of different symptoms. Thus, our research emphasizes the importance of a multidisciplinary approach to caring for post-acute COVID-19 patients, involving specialists from various medical fields to comprehensively address the different needs of patients.

Women aged 41 to 60 formed the highest proportion of subjects in our post-acute COVID-19 group. This is in line with an Italian cross-sectional study on 303 subjects, in which a structured interview on sequelae of COVID-19 at 1 year was administered by phone by trained medical staff^27^ and with the Danish’s study.^21^

The acute COVID-19 group, instead, consisted mostly of people aged 18 to 40 and this is in line with previous studies.^21,26^

Analyzing the persistence of symptoms in relation to participants’ characteristics, the probability of having post-acute COVID-19 was significantly higher in women and in those with a BMI ≥ 25, but also in patients older than 50 years and in those who were hospitalized during the acute phase of the disease as reported by others.^17,28,31^ We also noted that the pre-existing clinical conditions significantly associated with persistence of symptoms were autoimmune diseases, anxiety and allergies.

Various explanations, including hormonal factors and intrinsic personal factors like heightened sensitiveness, have been suggested for the increased susceptibility of women to post-acute COVID-19, contributing to a worse health experience.^33-35^

These findings suggest that gender must be taking into account in the management of post-acute COVID-19.

Post-acute COVID-19 is thought to be a result of persistent inflammation, unresolved tissue damage or delayed clearance of viral protein or RNA.^2^ Thus, overweight and obesity, which is recognized as a state of low-grade inflammation, might contribute to post-acute COVID-19.^36^ Moreover, also older age was reported to be associated with having persistent inflammation in post-acute COVID-19.^36^

As expected, COVID-19 hospitalized patients, in comparison with non-hospitalized patients, had a higher probability to develop post-acute COVID-19 because they had a more severe COVID-19.^37^ Actually, the existing literature report that the occurrence of post-acute COVID-19 is approximated to be between 10 and 30% among non-hospitalized patients, and between 50 and 70% among hospitalized patients,^38^ confirming our findings on a higher probability to develop post-acute COVID-19 for patients hospitalized during the acute phase of the disease.

To the best of our knowledge, this is one of the few studies reporting a significant association between previous autoimmune diseases and odds of post-acute-COVID-19. It was previously suggested that patients with COVID-19 develop multiple types of autoantibodies^39^ so it might be speculated that patients with s high autoantibodies level due to an autoimmune disease have a higher probability of post-acute-COVID-19. Moreover, it is also well known that the immune response is usually stronger in women than men. This could lead to post-acute-COVID-19 and could help explain the higher odds of post-acute-COVID-19 in women.^40^

We found no association between diabetes mellitus and persistence of symptoms, like Bernas et al,^31^ nor with the number of comorbidities, in contrast to Stavem et al.^25^

In general, analysis of FIS and 5Q-5D-3L questionnaires indicated that the post-acute COVID-19 group reported a higher impact of fatigue on their usual activities, and a worse quality of life, compared to the acute COVID-19 group. This is in line with the results reported by Hossain et al^26^ who, comparing the functional status of subjects who manifested symptoms during the acute phase and those with persistent symptoms, detected that the latter had more progressive functional limitation than the first. We observed a higher prevalence of post-acute COVID-19 individuals that showed a worsening in all 5 domains of 5Q-5D-3L (mobility, self-care, usual activities, pain/discomfort, and anxiety/depression) in comparison with acute COVID-19 group. In particular, the anxiety/depression domain was more prevalent in post-acute COVID-19 patients in comparison to acute-COVID-19 patients (47.9 vs 27%). Interestingly, Mazza et al^41^ found that patients not hospitalized during acute-COVID-19 showed a higher prevalence of anxiety and depression in comparison to those who were hospitalized (44.2 vs 32.3%).

These data further reinforce our findings, indicating that even individuals with a less severe acute disease experience significant sequelae. These results suggest to healthcare providers to recognize the importance of addressing mental health as part of the comprehensive care for individuals recovering from COVID-19. Mental health services, counseling, and support groups can be valuable resources in this context.

Overall, our findings bring attention on the management of post-acute COVID-19 in general practice, promoting a person-centered care and emphasizing the importance of actively listen to patients, understanding their experiences and perspectives, and addressing their needs, leading to more targeted and effective treatment strategies.

These may include, but are not limited to: design gradual and individually tailored exercise programs to help those with physical impairment and to promote weight loss; provide mental health support to address the psychological impact of post-acute COVID-19; promote the establishment of support groups post-acute COVID-19 patients can share experiences and strategies.

In addition, our study can enhance the understanding of health systems regarding the burden of this condition and can provide valuable insights that may be beneficial for addressing future pandemics or new variants of Sars-CoV-2.

### Strengths and Limitations

Our study has several strengths. Compared with other studies conducted in hospital out-patient units (hospitalized or non-hospitalized patients), this is one of the few studies in general practice, in which subjects with COVID-19 were invited by their GPs to voluntarily complete the survey, this limits the selection bias of subjects with more or less severe disease. In addition, this is the only study that collected self-reported data about the long-term consequences of COVID-19 on fatigue and quality of life, allowing participants to directly and freely explain their actual living conditions after COVID-19.

This study also has some limitations. The main limitation is the lack of a control group. Since post-COVID-19 symptoms are very similar to stress-related symptoms, a control group exposed to the same stress conditions would be useful to detect whether the persisting symptoms were in fact related to COVID-19. In addition, the self-reported nature of our survey implies that all the information about COVID-19 diagnosis and clinical course are not confirmed by a healthcare professional. This could lead to a misdiagnosis or the attribution of symptoms to COVID-19 when they may originate from alternative causes. Our survey was sent by-email to eligible participants among patients in charge of the participating GPs. Of them, those who were more sensitive to the topic of the research and those who were more familiar to the digital media, since the compilation of the survey was web-based, could be more likely to fill in the survey. In particular, older individuals could be under-represented.

In addition, the survey completion process may have discouraged individuals experiencing cognitive dysfunction or those who were no longer in poor health and lacked motivation to participate. This could affect the generalizability of the results.

The study’s retrospective nature raises the potential for recall bias, which may affect the reliability of the results. Since participants were asked to recall symptoms they experienced in the past, there is a chance of reporting inaccuracies. Thus, our results should be interpreted with caution. Moreover, unlike other studies, a limitation of our survey is that we were not able to distinguish between the severity of symptoms and the correlation between this severity and their duration.

## Conclusions

Our data showed that almost 29% of COVID-19 patients, mainly non-hospitalized during the acute phase of the disease, reported the persistence of symptoms beyond 4 weeks after COVID-19 diagnoses. The most persistent symptom in the post-acute phase was fatigue.

Our study provided data on post-acute COVID-19 symptoms in primary care setting where data is scanty and suggests the need for tailored personalized strategies, based on symptomatology and clinical profile, to improve the management of patients with post-acute COVID-19. With the ongoing pandemic and millions of individuals infected, the requirement of health care for sequelae of COVID-19 will continue to growth. Further data are needed to provide valuable insights that may be beneficial for addressing future pandemics on new variants of SARS-CoV-2.

## Supplemental Material

sj-docx-1-jpc-10.1177_21501319231222364 – Supplemental material for Prevalence and Predictors of Post-Acute COVID-19 Symptoms in Italian Primary Care Patients Click here for additional data file. Supplemental material, sj-docx-1-jpc-10.1177_21501319231222364 for Prevalence and Predictors of Post-Acute COVID-19 Symptoms in Italian Primary Care Patients by Andreana Foresta, Luisa Ojeda-Fernández, Claudia Augurio, Cecilia Guanziroli, Mauro Tettamanti, Giulia Macaluso, Paolo Lauriola, Alessandro Nobili, Maria Carla Roncaglioni and Marta Baviera in Journal of Primary Care & Community Health

sj-docx-2-jpc-10.1177_21501319231222364 – Supplemental material for Prevalence and Predictors of Post-Acute COVID-19 Symptoms in Italian Primary Care Patients Click here for additional data file. Supplemental material, sj-docx-2-jpc-10.1177_21501319231222364 for Prevalence and Predictors of Post-Acute COVID-19 Symptoms in Italian Primary Care Patients by Andreana Foresta, Luisa Ojeda-Fernández, Claudia Augurio, Cecilia Guanziroli, Mauro Tettamanti, Giulia Macaluso, Paolo Lauriola, Alessandro Nobili, Maria Carla Roncaglioni and Marta Baviera in Journal of Primary Care & Community Health

sj-docx-3-jpc-10.1177_21501319231222364 – Supplemental material for Prevalence and Predictors of Post-Acute COVID-19 Symptoms in Italian Primary Care Patients Click here for additional data file. Supplemental material, sj-docx-3-jpc-10.1177_21501319231222364 for Prevalence and Predictors of Post-Acute COVID-19 Symptoms in Italian Primary Care Patients by Andreana Foresta, Luisa Ojeda-Fernández, Claudia Augurio, Cecilia Guanziroli, Mauro Tettamanti, Giulia Macaluso, Paolo Lauriola, Alessandro Nobili, Maria Carla Roncaglioni and Marta Baviera in Journal of Primary Care & Community Health
