# Corrigendum to “Prevalence and Predictors of Post-Acute COVID-19 Symptoms in Italian Primary Care Patients”

**DOI:** 10.1177/21501319251341661

**Published:** 2025-05-11

Foresta A, Ojeda-Fernández L, Augurio C, et al. Prevalence and Predictors of Post-Acute COVID-19 Symptoms in Italian Primary Care Patients. *Journal of Primary Care & Community Health*. 2024;15. doi:10.1177/21501319231222364

Funding statement has been updated, and correct statement is as below:

**Funding**

This work was supported by Fondazione Cariplo (2021-4236 “The Lombardia long COVID Network for the implementation of best practices in the comprehensive management of long COVID (LLC-Network)), which covered the publication costs.

The article is updated online.

