# Supplementary material for: Prevalence and Predictors of Post-Acute COVID-19 Symptoms in Italian Primary Care Patients
Source: J Prim Care Community Health. 2024 Jan 3;15:21501319231222364. doi: 10.1177/21501319231222364 (PMC10768628; doi:10.1177/21501319231222364)
Supplement: sj-docx-1-jpc-10.1177_21501319231222364 – Supplemental material for Prevalence and Predictors of Post-Acute COVID-19 Symptoms in Italian Primary Care Patients [file sj-docx-1-jpc-10.1177_21501319231222364.docx]

**Annex 1. General Practitioners**

Abbiati Maria Cristina, Acciai Sofia, Adamo Daniela, Albanese Giuseppina Anna Rita, Andersen Sonia, Andreola, Angeli Martina, Anniboletti Sauro, Arabia Anna, Arioli, Asham Martin, Atanasio Stefano, Augurio Claudia, Badini Ernesto, Balzano Elisa, Barbera Valentina, Barcella, Basile Filomena, Belli, Bellini Valeria, Berbieri Fabio, Bergamelli Paolo, Bernasconi Mariagrazia, Bevilacqua, Bianchi Maurizio, Bianzini, Bianzini, Bianzini Claudio, Billi Claudio, Bisio Gabriella, Bizzi Paolo, Bocci Martina, Bombini Grazia, Bonato Santina, Bonetti, Bonsangue Sabrina, Bordone Pietro, Branzaglia, Buosi Maria Grazia, Cantù Vittorio, Cajani Francesca, Campagna Michele, Campiotti Sandro, Capaccioni Domenico, Capasso Barbato Bruno, Capitanio Vanessa, Capitanio Vanessa, Cappelli Claudio, Cappello Sonia, Caprioli, Caprioli Leonardo, Carminati Elena, Carminati Giacomo, Casati Gianpietro, Cascavilla Maria Teresa, Cascavilla Paolo, Cascini Valentina, Casella Antonio, Castoldi Riccardo, Cavazzuti, Cecola Carmine, Cefalalà Fancesco Maria,Centra Francesco, Ceriani, Chiarenza Salvatore, Chimenti Tullio, Cichello Pietro, Cileto Tiziana, Cilloni Domenico, Cimolino Tiziana, Cinelli Stefano, Ciocca Dorino, Cirioli Salvatore, Citelli, Cocchiola Margherita, Colombi Valentino, Comerio Martina, Corda Rosaria, Corti Elena, Costantino Biagio, Covizzi Rita Morena, Cremaschini Marco, Crippa Fabio, Crocilla Domenico, Cucchi, Cucurnia, Curcio Claudio, Dagheti Paola, De Angelis Angelo, De Angelis Angelo, De Gaspari Roberto, De Marco Tarcisio, De Rosa, De Sario Elisabetta, D'Ecclesia, Del Sonno Cristina, Della Corte, Della Morta, Di Lauro Laura, Di Leo, Di Martino Gennaro, Di Mattia Simona, Di Misa, Donadoni Silvano, Done, D'Ottavio Vincenzo, Dubini Silvia, Esposito, Fabbiano Stefano, Facchinetti, Fagnani Simona, Falsaperna Lorella, Fantoni Franco, Ferrari Antonella, Ferrari Erminia, Ferzi, Fossati Cesare, Fossati Roldano, Fraccari Alessandra, Frosali Laura, Fulgosi, Fusetti Giulia, Galvagno, Garifo, Ghiazza Barbara, Giacomini Valter, Giacosa, Giamba, Giannini Sergio, Gianotti Claudio, Gilardi Camilla, Giovannini Antonio, Giuffré Monica, Giuliana Matordes, Grattieri Roberto, Grimi Emanuela, Gullace Maria Angela, Iacono Carmen, Iemma Giuditta, Italiano Antonio, Iuliano Maurizio, La Cerenza Claudia, La Manna Piera, Lacerenza Claudia, Lamberti Giulia, Lauri Davide, Lazzarini Alessandro, Lazzaroni M.Chiara, Li Vecchi Daniela, Lisciandrano Dario, Locatelli Giuseppe, Lombardo Giulia, Lonardo Anna Camilla, Lorella Anita Schembri, Lupi Maria Antonietta, Luppino Francesca, Macchia Antonio, Magenta Paolo, Maggio Anna, Malmassari Chiara, Manera , Manneschi Mirko, Manuali Giampiero, Marasco Salvatore Danilo, Marenghi Carlo, Mariani Mariantonella, Marinini Alessio, Maris Nituleacililiana Gabriela, Markidis, Massinelli Angelo, Mastropietro, Matarazzo Maria, Matrone Daniela, Mele Antonella, Melidoni Rosa, Melluso Angelo, Melluso Elvira, Melucci Domenico, Meroni Laura, Messina Cristiano, Messina Enrico, Miano Paolo, Mila prati, Modica, Molteni Nicoletta, Montanari Valeria, Montemaggiore Franco, Montessori, Monti Massimo, Monticelli Pietro, Mor Luigi, Morales Filomeno, Moschetti Ivan , Munizza Ritaines, Nasi, Natale Domenico, Natalini Marco, Negri Luca, Nobili Stefano, Occhipinti, Olivieri Michele, Oreste Angelica, Orlacchio, Paccagnini Ottavia, Pacciani Mara, Pagano Alfonso, Palladino Michele, Parini, Parravicini Lorena, Patané Antonio, Pavarani Pietro, Pazzarelli, Pecora Nicola, Pedrocchi Luca, Pedroli William, Perugini, Perutzu Josetta, Petronio Salvatore, Petrucci Alessandro, Piazza, Picciallo Raguso Graziamaria, Pieralisi Fernando, Pisani Marina, Pisani Marina, Piscopiello Antonella, Platania, Poeta, Polidori Vincenzo, Polini, Prati Mila, Pronesti, Prussi, Punghellini Matteo, Putaggio Rosalba, Quagliata, Quilichini Mauro, Quitadamo Michelina, Rabbione Roberto, Radici Gianluigi, Raimondi Alessandra, Reali Fabrizio, Riva Anna, Rizzardi Nicola, Rizzi, Rizzo Maria, Rizzo Raffaele Francesco, Roberto Mara, Rocca Cristina, Rosa Enrico, Rosato, Rossi, Rotasperti, Rozzi Mara, Ruggeri Mario, Sangiorgio Nicola, Santagostino Sergio, Santamaria Vincenzo, Santini Guido, Scarduelli, Scurati, Serafini Alice, Seratoni Remo, Sertinean Natalia, Silvestri Marcello, Simonazzi, Simonich, Sist Giorgia, Spataro, Speziale Bruno, Spinelli Laura, Spreafico Alessio, Stanojevic Miroslav, Stillitano Giuseppe, Stroppa Stefania, Tamborini Ugo, Tesini Roberto, Tironi Marco, Tofani, Toniolo Alberto, Tozzi Antonio, Vece Angelo, Vegetti Leonardo, Verrengia Giovanni, Vindigni, Vullo Serafino, Zaghi Eva, Zampini Sara, Zancan, Zini, Zuccari Daniele.
