# Supplementary material for: Prevalence and Predictors of Post-Acute COVID-19 Symptoms in Italian Primary Care Patients
Source: J Prim Care Community Health. 2024 Jan 3;15:21501319231222364. doi: 10.1177/21501319231222364 (PMC10768628; doi:10.1177/21501319231222364)
Supplement: sj-docx-2-jpc-10.1177_21501319231222364 – Supplemental material for Prevalence and Predictors of Post-Acute COVID-19 Symptoms in Italian Primary Care Patients [file sj-docx-2-jpc-10.1177_21501319231222364.docx]

**Annex 2. Survey**

**Long Covid Survey in Medicina Generale**

❏ Ho letto e accetto l’**Informativa al trattamento dei dati personali**

**Data di compilazione: --/--/-----**

**Cara/o paziente, La ringraziamo per aver dato la disponibilità a partecipare a questa indagine. Qui di seguito le chiediamo di rispondere ad alcune semplici domande seguito le chiediamo di rispondere ad alcune semplici domande**

| **Informazioni socio-demografiche e fattori di rischio** | |
| --- | --- |
| Data di nascita | **__ / __ / ______** |
| Sesso | □ M  □ F |
| Comune di residenza | _________ |
| Scolarità | □ Licenza media inferiore  □ Licenza media superiore  □ Laurea |
| Peso | _____ kg |
| Altezza | _____ cm |
| Indice di massa corporea | *Calcolo automatico* |
| Fumatore | □ No  □ Si  □ Ex-fumatore |
| **Informazione sullo stato di salute prima dell’infezione** | |
| Malattie cardiache | □ No  □ Si  Se sì specificare ________ |
| Ictus cerebrale | □ No  □ Si |
| Ipertensione arteriosa | □ No  □ Si |
| Colesterolo alto | □ No  □ Si |
| Diabete | □ No  □ Si |
| Anemia | □ No  □ Si |
| Malattie respiratorie croniche | □ No  □ Si  Se sì specificare ________ |
| Malattie gastrointestinali | □ No  □ Si  Se sì specificare ________ |

| Malattie del fegato | □ No  □ Si  Se sì specificare ________ |
| --- | --- |
| Malattie del rene | □ No  □ Si  Se sì specificare ________ |
| Malattie autoimmuni | □ No  □ Si  Se sì specificare ________ |
| Allergie | □ No  □ Si  Se sì specificare ________ |
| Tumore | □ No  □ Si  Se sì specificare ________ |
| Ansia | □ No  □ Si |
| Depressione | □ No  □ Si |
| Altre patologie | ________________ |
| **Diagnosi di COVID-19 ed evoluzione della malattia** | |
| Ha eseguito un tampone molecolare per COVID-19? | □ No  □ Si  *Se la risposta è Si:* |
| Data del primo tampone con esito positivo: | -- / -- / -----  □ Non so |
| Data del primo tampone con esito negativo, se effettuato: | -- / -- / -----  □ Non so  □ Non effettuato |
| È stato ricoverato per COVID-19? | □ No  □ Si  *Se la risposta è Si:* |
| Data di ricovero e dimissione: | Data ricovero: -- / -- / -----  □ Non so  Data dimissione: -- / -- / -----  □ Non so |
| Ha avuto la necessità di un supporto di ossigeno in ospedale? (mascherina o casco) | □ No  □ Si |
| È stato ricoverato in terapia intensiva? | □ No  □ Si |
| È stato intubato? | □ No  □ Si |
| Dopo la dimissione, è tornato presso il suo domicilio o presso una struttura assistenziale per la riabilitazione? | □ Domicilio  □ Struttura assistenziale |
| Ha avuto la necessità di un supporto di ossigeno a domicilio dopo la dimissione? | □ No  □ Si |
| **Sintomi eventualmente presenti durante la malattia da COVID-19 e loro durata** | |
| Febbre (>37.5°C) | □ No  □ Si  *Se la risposta è Si:* |
| Per quanto tempo? | □ ____ giorni  □ ____ settimane |
| Tosse | □ No  □ Si  *Se la risposta è Si:* |
| Per quanto tempo? | □ ____ giorni  □ ____ settimane  □ ____ mesi  □ Ancora in corso |
| Mal di testa | □ No  □ Si  *Se la risposta è Si:* |
| Per quanto tempo? | □ ____ giorni  □ ____ settimane  □ ____ mesi  □ Ancora in corso |
| Dolori articolari | □ No  □ Si  *Se la risposta è Si:* |
| Per quanto tempo? | □ ____ giorni  □ ____ settimane  □ ____ mesi  □ Ancora in corso |
| Dolori muscolari | □ No  □ Si  *Se la risposta è Si:* |
| Per quanto tempo? | □ ____ giorni  □ ____ settimane  □ ____ mesi  □ Ancora in corso |
| Difficoltà respiratoria | □ No  □ Si  *Se la risposta è Si:* |
| Per quanto tempo? | □ ____ giorni  □ ____ settimane  □ ____ mesi  □ Ancora in corso |
| Stanchezza/Debolezza muscolare | □ No  □ Si  *Se la risposta è Si:* |
| Per quanto tempo? | □ ____ giorni  □ ____ settimane  □ ____ mesi  □ Ancora in corso |
| Perdita di appetito | □ No  □ Si  *Se la risposta è Si:* |
| Per quanto tempo? | □ ____ giorni  □ ____ settimane  □ ____ mesi  □ Ancora in corso |
| Perdita dell’olfatto (anosmia) | □ No  □ Si  *Se la risposta è Si:* |
| Per quanto tempo? | □ ____ giorni  □ ____ settimane  □ ____ mesi  □ Ancora in corso |
| Perdita del gusto (ageusia) | □ No  □ Si  *Se la risposta è Si:* |
| Per quanto tempo? | □ ____ giorni  □ ____ settimane  □ ____ mesi  □ Ancora in corso |
| Alterazioni uditive/perdita temporanea dell’udito | □ No  □ Si  *Se la risposta è Si:* |
| Per quanto tempo? | □ ____ giorni  □ ____ settimane  □ ____ mesi  □ Ancora in corso |
| Vertigini | □ No  □ Si  *Se la risposta è Si:* |
| Per quanto tempo? | □ ____ giorni  □ ____ settimane  □ ____ mesi  □ Ancora in corso |
| Nausea/Vomito | □ No  □ Si  *Se la risposta è Si:* |
| Per quanto tempo? | □ ____ giorni  □ ____ settimane  □ ____ mesi  □ Ancora in corso |
| Diarrea | □ No  □ Si |
| Rinite (naso che cola) | □ No  □ Si  *Se la risposta è Si:* |
| Per quanto tempo? | □ ____ giorni  □ ____ settimane  □ ____ mesi  □ Ancora in corso |
| Tachicardia/Palpitazioni | □ No  □ Si  *Se la risposta è Si:* |
| Per quanto tempo? | □ ____ giorni  □ ____ settimane  □ ____ mesi  □ Ancora in corso |
| Disturbi del sonno | □ No  □ Si  *Se la risposta è Si:* |
| Per quanto tempo? | □ ____ giorni  □ ____ settimane  □ ____ mesi  □ Ancora in corso |
| Disturbi della memoria | □ No  □ Si  *Se la risposta è Si:* |
| Per quanto tempo? | □ ____ giorni  □ ____ settimane  □ ____ mesi  □ Ancora in corso |
| Difficoltà a concentrarsi o a svolgere attività lavorative o di studio? | □ No  □ Si  *Se la risposta è Si:* |
| Per quanto tempo? | □ ____ giorni  □ ____ settimane  □ ____ mesi  □ Ancora in corso |
| Sensazione di annebbiamento | □ No  □ Si  *Se la risposta è Si:* |
| Per quanto tempo? | □ ____ giorni  □ ____ settimane  □ ____ mesi  □ Ancora in corso |
| Manifestazioni cutanee tipo rossore o prurito | □ No  □ Si  *Se la risposta è Si:* |
| Per quanto tempo? | □ ____ giorni  □ ____ settimane  □ ____ mesi  □ Ancora in corso |
| Perdita di capelli | □ No  □ Si  *Se la risposta è Si:* |
| Per quanto tempo? | □ ____ giorni  □ ____ settimane  □ ____ mesi  □ Ancora in corso |
| Congiuntivite | □ No  □ Si  *Se la risposta è Si:* |
| Per quanto tempo? | □ ____ giorni  □ ____ settimane  □ ____ mesi  □ Ancora in corso |
| Altri sintomi | ___________ |
| Per quanto tempo? (per ogni sintomo) | □ ____ giorni  □ ____ settimane  □ ____ mesi  □ Ancora in corso |
| Ha assunto dei farmaci specifici per trattare uno di questi sintomi? |  |
| Se sì, indicare quale | ___________ |
| Ha aumentato il numero di farmaci cronici che assume? | □ No  □ Si |
| Quanti farmaci cronici prendeva prima del COVID-19? | __ |
| Ad oggi quanti farmaci prende al giorno? | __ |
| È mai stato ricoverato per altri motivi dopo il tampone negativo? | □ No  □ Si |
| Se sì, indicare quale | ___________ |
| **Compilare questi brevi questionari sulla fatica e sulla qualità di vita** | |
| **Questionario sulla fatica**  Di seguito è riportato un elenco di affermazioni che descrivono come la fatica può causare problemi nella vita delle persone.  Si prega di leggere attentamente ogni affermazione e inserire una "X" nella casella che indica meglio quanto la sensazione di affaticamento le ha causato problemi durante la giornata di oggi.  Selezioni UNA casella per ogni affermazione e non ne salti nessuna. | 1. A causa della stanchezza, mi sento meno vigile.  □ Nessun problema  □ Piccoli problemi  □ Problemi moderati  □ Grandi problemi  □ Problemi enormi  2. A causa della stanchezza, devo ridurre il mio carico di lavoro e le mie responsabilità  □ Nessun problema  □ Piccoli problemi  □ Problemi moderati  □ Grandi problemi  □ Problemi enormi  3. A causa della stanchezza, sono meno motivato a fare qualsiasi cosa che richieda uno sforzo fisico  □ Nessun problema  □ Piccoli problemi  □ Problemi moderati  □ Grandi problemi  □ Problemi enormi  4. A causa della stanchezza, ho problemi ad effettuare uno sforzo fisico per un lungo periodo  □ Nessun problema  □ Piccoli problemi  □ Problemi moderati  □ Grandi problemi  □ Problemi enormi  5. A causa della stanchezza, trovo difficoltà a prendere una decisione  □ Nessun problema  □ Piccoli problemi  □ Problemi moderati  □ Grandi problemi  □ Problemi enormi  6. A causa della stanchezza, sono meno capace di finire dei compiti che richiedono una riflessione.  □ Nessun problema  □ Piccoli problemi  □ Problemi moderati  □ Grandi problemi  □ Problemi enormi  7. A causa della stanchezza, sento un rallentamento della mia capacità di pensare  □ Nessun problema  □ Piccoli problemi  □ Problemi moderati  □ Grandi problemi  □ Problemi enormi  8. A causa della stanchezza, devo eliminare l’attività fisica  □ Nessun problema  □ Piccoli problemi  □ Problemi moderati  □ Grandi problemi  □ Problemi enormi |
| **EQ-5D-3L**  **Questionario sulla qualità di vita** Quale delle seguenti affermazioni descrive meglio il suo stato di salute oggi, segnando con una crocetta una sola casella di ciascun gruppo. | **Capacità di Movimento**  □ Non ho difficoltà nel camminare  □ Ho qualche difficoltà nel camminare  □ Sono costretto/a a letto  **Cura della Persona**  □ Non ho difficoltà nel prendermi cura di me stesso/a  □ Ho qualche difficoltà nel lavarmi o vestirmi  □ Non sono in grado di lavarmi o vestirmi  **Attività Abituali (per es. lavoro, studio, lavori domestici, attività familiari o di svago)**  □ Non ho difficoltà nello svolgimento delle attività abituali  □ Ho qualche difficoltà nello svolgimento delle attività abituali  □ Non sono in grado di svolgere le mie attività abituali  **Dolore o Fastidio**  □ Non provo alcun dolore o fastidio  □ Provo dolore o fastidio moderati  □ Provo estremo dolore o fastidio  **Ansia o Depressione**  □ Non sono ansioso/a o depresso/a  □ Sono moderatamente ansioso/a o depresso/a  □ Sono estremamente ansioso/a o depresso/a  Per aiutarla ad esprimere il suo stato di salute attuale, vorremmo che indicasse su una scala da 0 a 100 (sulla quale il migliore stato di salute immaginabile è contrassegnato dal numero 100 ed il peggiore dallo 0) quale è, secondo lei, il livello del suo stato di salute oggi. |
| ***Grazie per la sua disponibilità e il suo contributo!*** | |

| **Informazione per contatto** | |
| --- | --- |
| *Per essere informato sui i risultati della presente indagine a cui ha partecipato e per permettere al suo Medico di ricontattarla, La invitiamo a lasciarci i suoi contatti.* | |
| **Nome e cognome** |  |
| **Indirizzo email:** |  |
| **Telefono:** |  |
| **Medico di Medicina Generale:** |  |
