# Supplementary material for: Prevalence and Predictors of Post-Acute COVID-19 Symptoms in Italian Primary Care Patients
Source: J Prim Care Community Health. 2024 Jan 3;15:21501319231222364. doi: 10.1177/21501319231222364 (PMC10768628; doi:10.1177/21501319231222364)

**SUPPLEMENTARY MATERIAL**

**Prevalence and duration of COVID-19 symptoms in primary care patients: An online-based survey**

**Figure 1S.** Study Flowchart

People invited to take part in the survey

From 01/10/2021 – 31/12/2022

N = 1748

Included Participants
N=1721

**Excluded**

N=5

Age < 18 ys

**Excluded**

N=22

No online informed consent form

**Excluded**

N=613

Did not complete the survey

Survey Analysed
**N=1108**

**Aymptomatic subjects**

N=39

Did not report symptoms

**Acute-COVID-19**

N=320

Symptoms 0-4 weeks

**Post-acute-COVID-19**

N=749

Symptoms >4 weeks

**Table 1S.** Prevalence and duration of each symptom in the whole study period and after four weeks

| **Symptoms** | **Total study period**  **Number** (%) **Mean** (days) ± SD  **Median** (days) **[q1, q3]** | **After 4weeks**  **Number** (%)  **Mean** (days) ± SD,  **Median** (days) **[q1, q3]** |
| --- | --- | --- |
| Fever (>37.5°C) | 672 (62.86) | 0 (0) |
|  | 3.52 ± 3.18 3 [2, 4] | - |
| Cough | 668 (62.49) | 53 (5.0) |
|  | 13.91 ± 26.25  7 [5, 14] | 68.89 ± 69.03,  60 [35, 60] |
| Headache | 575 (53.79) | 33 (3.1) |
|  | 14.10 ± 50.62  4 [2, 7] | 150.97±156.23  84 [60, 180] |
| Joint pain | 585 (54.72) | 63 (5.9) |
|  | 29.64 ± 95.98  6 [3, 14] | 209.2±218.7  95 [56, 281] |
| Muscle Pain | 584 (54.63) | 68 (6.4) |
|  | 30.90 ± 100.51  6 [3, 14] | 206.09±225.01  92.5 [60, 254.5] |
| Breathing difficulties | 242 (22.64) | 48 (4.5) |
|  | 43.53 ± 103.48  10 [3, 25] | 175.52±173.13  90 [60, 267.5] |
| Fatigue | 818 (76.52) | 178 (16.7) |
|  | 57.52 ± 431.04  13.50 [5, 28] | 217.02±884.43  82.5 [55,180] |
| Loss of appetite | 296 (27.69) | 23 (2.2) |
|  | 17.03 ± 49.90  7 [4, 12] | 126.61±137.95  84 [53, 128] |
| Anosmia | 318 (29.75) | 56 (5.2) |
|  | 87.27 ± 711.75  10 [5, 21] | 433.04±1630.97  60 [54, 255] |
| Ageusia | 298 (27.88) | 40 (3.7) |
|  | 68.66 ± 689.22  8 [5, 21] | 436.65±1835.99  60 [49, 200] |
| Hearing impairment | 28 (2.62) | 8 (0.7) |
|  | 67.72 ± 113.31  15 [4, 60] | 190.75±135.86  180 [75, 250] |
| Dizziness | 114 (10.66) | 21 (2.0) |
|  | 59.20 ± 158.09  5 [3, 28] | 262.52±269.65  212 [42, 390] |
| Nausea | 147 (13.75) | 9 (0.8) |
|  | 21.66 ± 79.27  3 [2, 7] | 271.33±194.59  292 [70, 375] |
| Diarrhea | 194 (18.15) | - |
| Rhinitis | 534 (49.95) | 29 (2.7) |
|  | 35.23 ± 514.24  5 [4, 9.50] | 514.52±2156.21  70 [53, 169] |
| Tachycardia | 133 (12.44) | 43 (4.0) |
|  | 87.02 ± 169.26  14 [5, 60] | 236.44±222.79  120 [60, 374] |
| Sleep disorders | 225 (21.05) | 70 (6.5) |
|  | 89.85 ± 167.19  15 [7, 60] | 239.9±216.43  180 [60, 364] |
| Memory impairment | 128 (11.97) | 72 (6.7) |
|  | 215.91 ± 430.01  91 [21, 259] | 308.4± 493.17  180 [90, 389.5] |
| Concentration impairment | 323 (30.22) | 100 (9.4) |
|  | 74.24 ± 138.51  14.50 [7, 60] | 193.29± 183.22  103 [60, 280] |
| Feeling of fogginess | 137 (12.82) | 47 (4.4) |
|  | 75.51 ± 147.26  15 [4, 60] | 182.68±196.04  90 [52, 229] |
| Skin manifestations | 89 (8.33) | 25 (2.3) |
|  | 68.16 ± 168.81  10 [4, 30] | 198.72±257.98  90 [30, 229] |
| Hair loss | 100 (9.35) | 47 (4.4) |
|  | 142.35 ± 200.54 60 [21, 146] | 218.17±221.21  95 [60, 360] |
| Conjunctivitis | 57 (5.33) | 9 (0.8) |
|  | 49.96 ± 134.08 7 [4, 21] | 268.11±243.9  180 [60, 436] |

**Figure 2S.** Percentages of acute COVID-19 (A) and post-acute COVID-19 subjects (B), stratified according to age and sex.

**A**

**
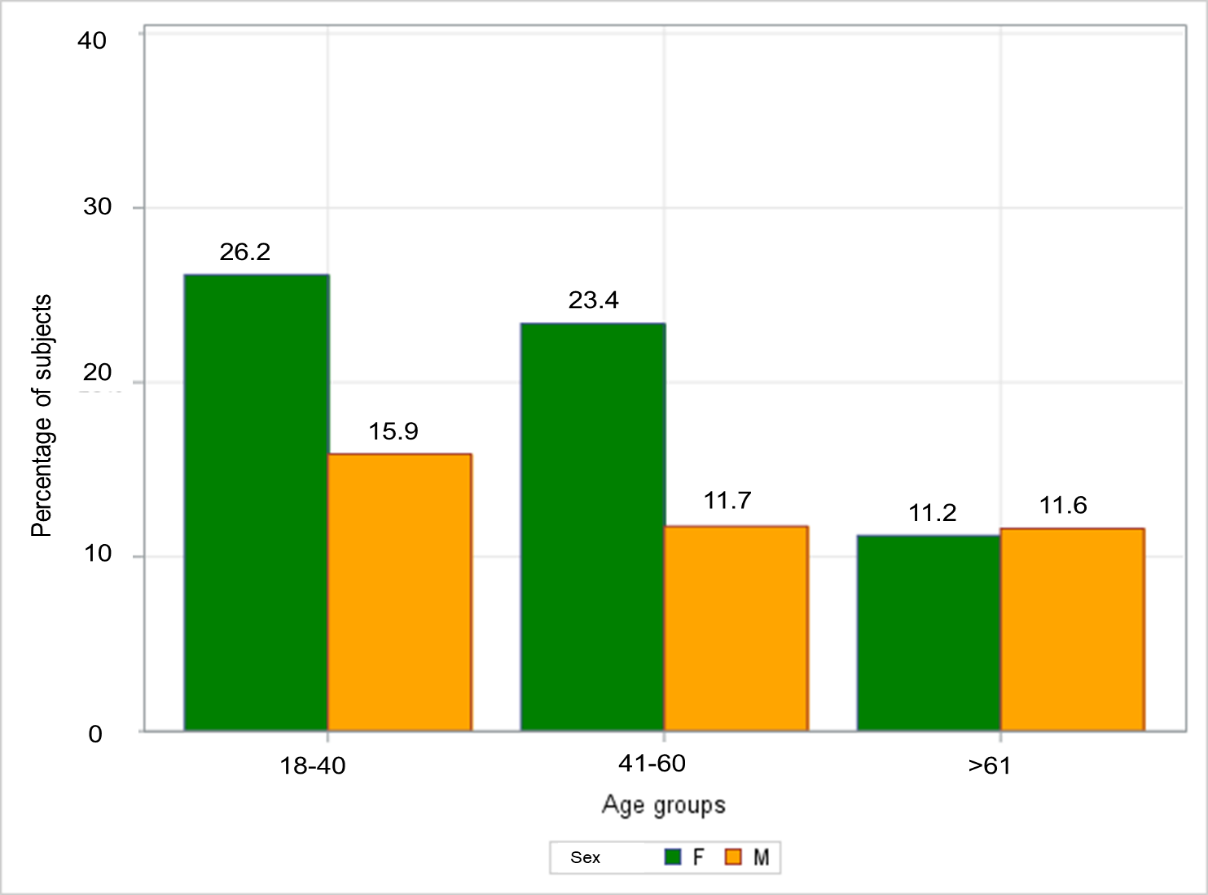
**

**B**


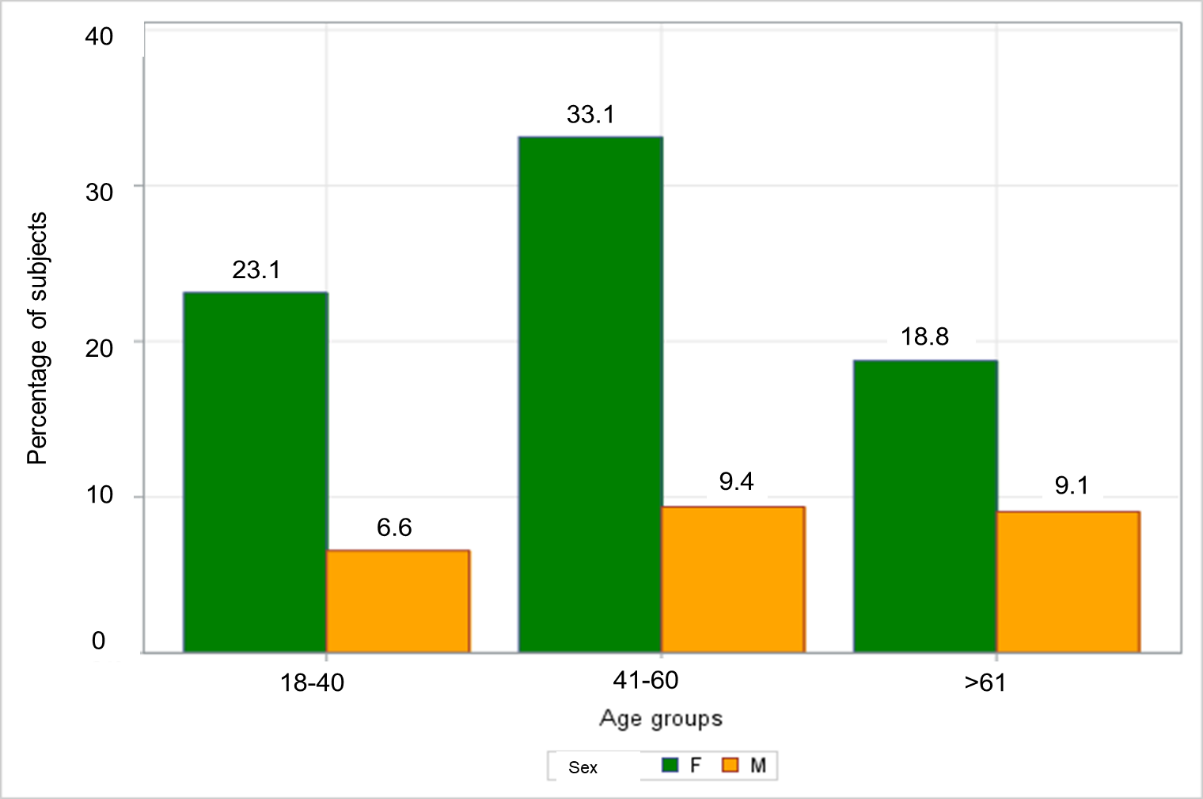

Supplement: sj-docx-3-jpc-10.1177_21501319231222364 – Supplemental material for Prevalence and Predictors of Post-Acute COVID-19 Symptoms in Italian Primary Care Patients [file sj-docx-3-jpc-10.1177_21501319231222364.docx]
